# Indocyanine Green-Assisted Internal Limiting Membrane Peeling in Macular Hole Surgery: A Meta-Analysis

**DOI:** 10.1371/journal.pone.0048405

**Published:** 2012-11-07

**Authors:** Yan Wu, Wei Zhu, Ding Xu, Yan-Hong Li, Jun Ba, Xiao-Long Zhang, Fang Wang, Jing Yu

**Affiliations:** 1 Department of Ophthalmology, Affiliated Tenth People's Hospital of Tongji University, Shanghai, China; 2 Department of First Clinical Medical College, Nanjing Medical University, Nanjing, Jiangsu, China; Sun Yat-sen University, China

## Abstract

**Background:**

The opinion of application of indocyanine green (ICG) in the macular hole surgery was contradictory. Here we conducted a meta-analysis to evaluate the effect of in internal limiting membrane (ILM) peeling for macular hole surgery.

**Methods and Findings:**

We searched electronic databases for comparative studies published before July 2012 of ILM peeling with and without ICG. Twenty-two studies including 1585 eyes were included. Visual acuity (VA) improvement, including the postoperative rate of ≥20/40 VA gained (OR, 0.65; 95% CI, 0.43 to 0.97; *P* = 0.033) and increased LogMAR (WMD, −0.09; 95% CI, −0.16 to −0.02; *P* = 0.011), was less in the ICG group. The risk of visual field defects was greater in the ICG group than in the non-ICG group. There was no significant difference in the rate of anatomical outcomes between ILM peeling procedures performed with and without ICG. RPE changes and other postoperative complications were not significantly different between the ICG and non-ICG groups. An additional analysis showed that the VA improvement of the ICG group was less than the non-ICG group only within the first year of follow up. A subgroup analysis showed that the rate of VA improvement was lower in the ICG group than in other adjuncts group. A higher rate of secondary closure and less VA improvement were observed in a high proportion (>0.1%) of the ICG group. A sensitivity analysis after the randomized-controlled trials were excluded from the meta-analysis demonstrated no differences compared with the overall results.

**Conclusions:**

This meta-analysis demonstrated that there is no evidence of clinical superiority in outcomes for ICG-assisted ILM peeling procedure over the non-ICG one. The toxicity of ICG should be considered when choosing the various staining methods.

## Introduction

Since the removal of the internal limiting membrane (ILM) was initially performed during macular hole (MH) surgery, contradictory opinions have been reported about its contribution to the procedure [1]. Considering the pathophysiology of MHs, ILM peeling has been regarded as a hopeful surgical approach for improving the anatomical outcome of MH surgery [2]. Compared with a closure rate of 69% without ILM peeling [3], the closure rate (87.8%–100%) [4]–[6] for MH surgery with ILM peeling is higher. However, the ILM is an achromatic thin membrane, and its nonvisibility makes this maneuver a challenge for surgeons. Indocyanine green (ICG) is a tricarbocyanine dye that is used in ophthalmology in many countries when treating chorioretinal disorders [7], angiography [8], cataracts [9] and corneal vascularization [10]. The use of ICG to improve ILM visualization made ILM peeling popular in MH surgery. In general, the use of vitrectomy, adjuncts (e.g., growth factor and autologous serum), a postoperative face-down posture and ILM peeling improved the anatomical and functional outcomes of MH surgery [11], [12].

The initial enthusiasm for intravitreal ICG application was dampened when some studies claimed possible toxicity and adverse effects of ICG in the management of MHs. Sippy BD and associates [13] showed that ICG was potentially toxic to retinal pigment epithelium (RPE) cells. In addition, several studies have reported different postoperative complications. Engelbrecht and associates [11] reported that the higher incidence of RPE changes was corrected with ICG-assisted ILM peeling. However, visual field defects [14] and worse visual acuity (VA) [1] were reported by other researchers as the results of ICG toxicity or operative trauma induced by ICG. Conversely, some studies have shown that both the anatomical and functional outcomes are better in ICG-stained eyes than unstained eyes [15], [16]. Overall, no consensus opinion exists on the use and application of ICG.

In recent years, several studies have reported inconsistent results. We conducted a meta-analysis to evaluate the use of ICG for ILM peeling in MH surgery. The anatomical outcomes, functional outcomes and postoperative complications between the ICG group and the non-ICG group were evaluated.

## Materials and Methods

This meta-analysis was conducted according to the Preferred Reporting Items for Systematic Reviews and Meta-Analyses (PRISMA) guidelines. [17] No protocol exists for this current systematic review.

### 1. Search strategy

Electronic databases were searched to retrieve related studies published before July 2012 with the Medical Subject Heading (MeSH) keywords “macular hole”, “indocyanine green”, “comparative study” and combinations of the words in addition to the keywords “internal limiting membrane”, “ICG”, “dye”, “stain” and “vitrectomy”. The citations of the identified articles were examined for additional studies. The language was restricted to English.

### 2. Inclusion criteria

The articles were considered eligible if the studies met the following inclusion criteria: (1) comparative studies; (2) contained at least two groups: with and without the application of ICG; (3) only macular hole patients were included, and ILM peeling was conducted in case and control groups; (4) at least one of the outcomes of interest were included.

### 3. Data extraction

The data were extracted independently by two reviewers (Y. Wu and W. Zhu) and were rechecked after the first extraction. Any disagreement regarding eligibility during the extraction was discussed by the two reviewers and resolved. The information extracted from each study included the first author, year, country, trial type, age, gender, preoperative best corrected visual acuity (BCVA), follow-up time, symptom duration, osmolarity and the solvents used. The outcomes of interest that were extracted included the following: the anatomical outcome, including the rates of primary, secondary and final closure; the functional outcome, including the rate of VA gain ≥20/40, VA improvement ≥2 lines, improved VA and increased LogMAR value; the postoperative complications, including the risk of RPE changes, retinal detachment, retinal tears, visual field defects, macular edema and optic nerve fiber layer changes.

### 4. Assessment of methodology quality

The quality of the included studies was assessed using the US Preventive Services Task Force grading system [18], the Downs and Black quality assessment method [19] and the Newcastle-Ottawa Scale (NOS) [20]. The Newcastle-Ottawa Scale (NOS) was used to evaluate only non-RCTs and the selection, comparability and outcome or exposure for cohort or case-control studies. The maximum for selection was 4 *, for comparability was 2 * and for outcome or exposure was 3 *. The maximum NOS score was 9 *, and the studies with ≥6 * were considered to have relatively higher quality.

### 5. Statistical analysis

The meta-analysis was conducted using the Stata software package (version 11.0; Stata Corp., College Station, TX). For dichotomous variables, the odds ratios (ORs) were measured with 95% confidence intervals (CIs), while the weighted mean difference (WMD) was measured with the 95% CIs for continuous variables. Both ORs and WMDs were considered statistically significant at the *P*<0.05 level. Statistical heterogeneity among studies was evaluated with the *χ^2^* and I^2^ tests. Both a fixed-effects model and a random-effects model were used to obtain summary ORs or WMDs. In the absence of heterogeneity between groups, the fixed-effects model and the random-effects model provided concordant results, and the random-effects model was employed only when heterogeneity was significant.

To deal with the “zero cells” for the number of events of interest, which created problems in the ORs measure and its standard error of the treatment effect, the value 0.5 was added in each cell of the 2×2 table. If no event or all events were present for both the case and control groups, the study was dropped from the meta-analysis [21]. The following subgroup analyses were performed: (1) the outcomes of interest between unstained eyes or other stains in the control groups; (2) the outcomes of interest between high and low concentrations of ICG (ICG concentrations of ICG>1% and ≤1%, respectively) and the non-ICG group. A sensitivity analysis was conducted in which the RCTs were excluded to thereby determine the stability of the combined ORs or WMDs. A subgroup analysis and a meta-regression [22] were adopted to analyze the source of heterogeneity. Potential publication bias was estimated by both visually evaluating a funnel plot and the Egger test [23], [24].

## Results

### 1. Literature search

**Figure 1:**
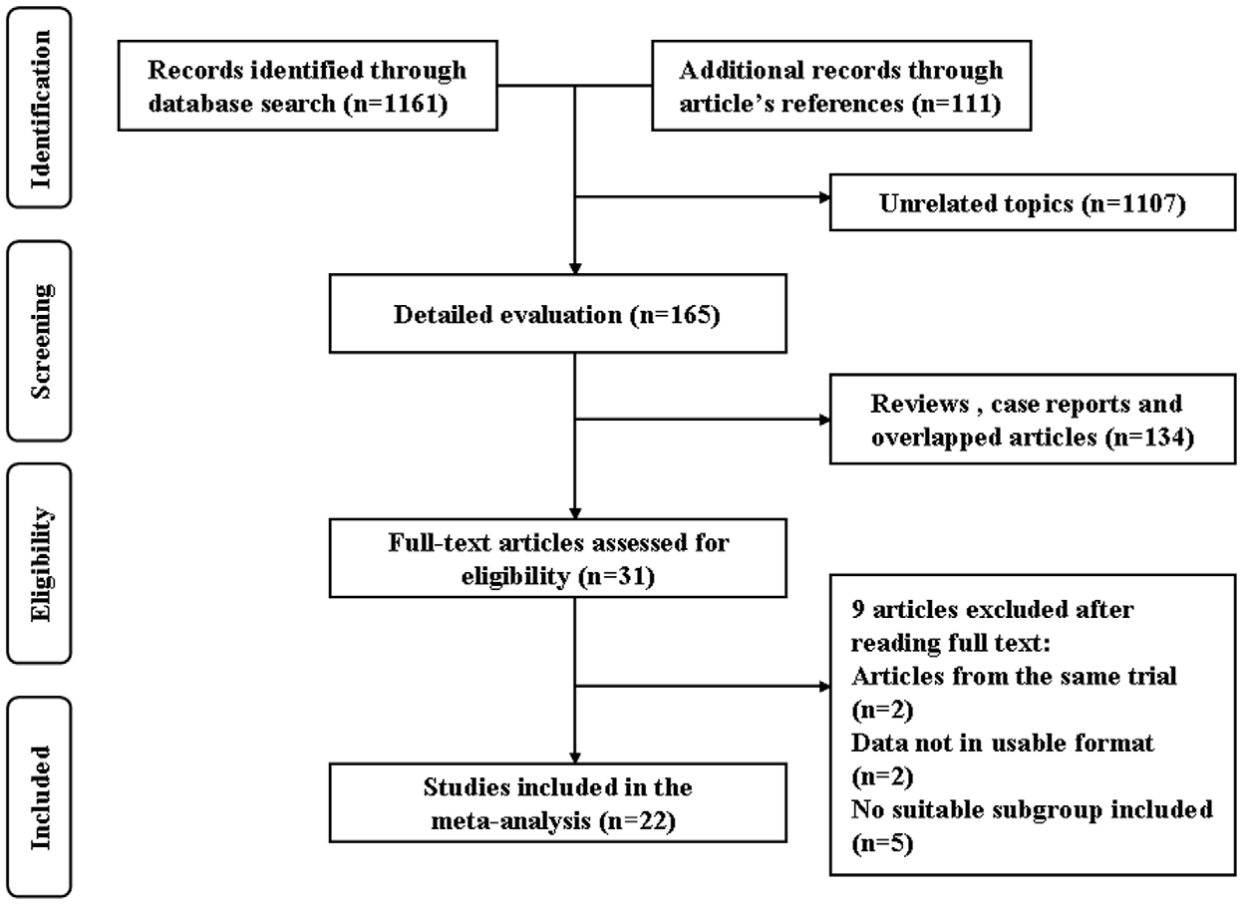
Search strategy flow diagram about with or without the use ICG in macular hole surgery.

A total of 1272 articles were initially identified; 1161 records were identified in the database search, and 111 records were found in article reference lists. Subsequently, 31 articles with full text that met the inclusion criteria were assessed. Three articles were from the same clinical trial, and the most eligible article was chosen, 2 articles did not contain usable data, and 5 articles did not contain suitable subgroups. A final total of 22 studies [6], [15], [16], [25]–[43] published from 2003 to 2011 were included in this meta-analysis. Figure 1 provides a flow diagram of the search results.

### 2. Characteristics and baseline of the included studies

In total, there were 1585 eyes included in this meta-analysis; 858 eyes were included in the ICG group, and 727 eyes were included in the non-ICG group. The characteristics of these included studies are shown in Table 1. The study design was retrospective in 18 studies, prospective in 1 study and randomized in 3 studies. Although 1 study was partially prospective, the portion comparing the ICG group with the non-ICG group was retrospective; it was therefore defined as retrospective [28]. The studies had the following geographic distribution: 9 in Asia, 7 in Europe, 3 in the US, 2 in Oceania and 1 international.

**Table 1:** Description of the characteristics of the included trials.

First author, Year	Country	Trial Type, LOE	Study Quality^b^					Matching^c^
			Doowns And Black Score	NOS Scale				
				Selection	Comparability	Expose	Total Score	
Brockmann T [27], 2011	Germany	Retrospective,4	14	***	**	*	******	1, 2, 3, 4, 5, 7
Shukla D [28], 2011	India	Retrospective^a^, 4	16	****	**	*	*******	1, 2, 3, 5, 7
Christensen UC [29], 2009	Denmark	RCT, 2	21	—	—	—	—	1, 5, 7
Schaal S [30], 2009	US	Retrospective, 3	15	***	*	**	******	1, 3, 5, 6, 7
Nakamura Y [31], 2007	Japan	Retrospective, 4	16	***	**	**	*******	1, 2, 3, 4, 5, 6, 7
Thompson JT [32], 2007	US	Retrospective, 4	15	**	**	**	******	1, 3, 5, 6
Nomoto H [33], 2008	Japan	Retrospective, 4	14	***	*	**	******	1, 2, 3, 5, 7
Nagai N [34], 2007	Japan	Retrospective, 4	14	***	**	*	******	1, 2, 3, 4, 5,
Beutel J [35], 2007	Germany.	RCT, 3	21	—	—	—	—	1, 2, 3, 5, 6, 7
Ferencz M [36], 2006	Hungary	Prospective, 3	15	***	**	*	******	1, 2, 3, 4, 5, 7
Kumagai K [37], 2006	Japan	Retrospective, 4	15	***	*	*	*****	1, 2, 4, 5, 6
Husson-Danan A [38], 2006	France	Retrospective, 4	16	***	*	**	******	3, 4, 6, 7
Lee KL [39], 2005	New Zealand	Retrospective, 4	14	***	**	*	******	1, 3, 5, 6, 7
Karacorlu M [40], 2005	Turkey	Retrospective, 4	14	***	*	*	*****	1, 2, 3, 4, 6, 7
Slaughter K [41], 2004	Australia	Retrospective, 4	15	**	*	**	*****	1, 2, 3, 4, 5, 6
Ando F [16], 2004	Japan	Retrospective, 3	16	***	**	**	*******	1, 2, 3, 4, 5, 6, 7
Lochhead J [15], 2004	UK	Retrospective, 4	14	****	*	*	******	1, 2, 3, 4, 5, 7
Sheidow TG [42], 2003	International	Retrospective, 4	16	***	**	**	*******	1, 3, 4, 5, 7
Horio N [43], 2004	Japan	RCT,2	20	—	—	—	—	1, 2, 3, 4, 5, 7
Eiko Tsuiki [25], 2007	Japan	Retrospective, 4	14	**	0	*	***	2, 3, 7
Kouki Fukuda [6], 2011	Japan	Retrospective, 4	15	**	**	*	******	1, 2, 3, 4, 5, 7
Mavrofrides E [26], 2006	US	Retrospective, 4	14	**	*	*	****	1, 2, 4, 5, 6, 7

— = no data provided; RCT = randomized-controlled trials; LOE = level of evidence.

a The part included in the partly retrospective study is retrospective.

b The study quality is evaluated by Downs and Black score and Newcastle-Ottowa Scale (NOS). The Downs and Black score for both RCT and non-RCT while NOS for RCT only.

c The matching factors are: (1) age, (2) gender, (3) macular hole type, (4) symptom duration, (5) preoperative visual acuity, (6) one surgeon, (7), follow-up time.

### 3. Quality assessment

Among the 9 trials conducted in Asia, 8 were from Japan. The level of evidence of each study was over 4, and the level of each RCT was at least 3. For the Downs and Blacks score, all studies were over 14, and all of the RCTs were over 20. Additionally, 15 of the 19 non-RCTs had scores ≥6 *. The lowest score was 3 * because its comparability and exposure scores were low. To determine whether related factors between the case and control groups were matched, all of the matching groups for each included study were listed. Among all of the studies, 12 of 22 trials contained more than 20 eyes in both the case and control groups. Idiopathic MHs were included in 13 trials, while mixed types were included in 2 trials. The baseline of each included trial is presented in Table 2. The outcomes examined were anatomical outcome, functional outcome and postoperative complications. The main outcomes in each study are listed in Table 3. The anatomical outcome was weighted by both primary and secondary closure rates, and the functional outcome was judged by the rate of gain of a VA≥20/40, VA improvement ≥2 lines and an increased LogMAR value. Several postoperative complications were reported, including RPE changes, retinal detachment and visual field defects. However, only the most important indexes are listed in the table, and several less important complications were recorded for a subsequent meta-analysis.

**Table 2:** Description of the baseline of included trials.

First author, Year	No. Eyes	Age (Y)	Gender (male/female)	Preoperative BCVA	Follow-Up	Symptom Duration	MH Type/Stage	ICG Concentration/Expose Time	Osmolarity (mOsm)	Solvent
Brockmann T [27], 2011^a^e	25(13∶12)	67.00±17.70/69.1±8.7	3∶10/3∶9	0.20±0.11/0.25±0.16	1 M	NA	NA/(S1b–S4)	0.1%/<20 s	NA	NA
Shukla D [28], 2011^a^^, ^ b	50(35∶15)	58.73/59.09	24∶26	0.19/0.19	≥6 M	NA	Idiopathic/(S3–4)	0.5%/30–60 s	NA	NA
Christensen UC [29], 2009^b^	78(35∶18)	66.9/66.6	8∶27/9∶9	50.5±5.9/49.9±6.5	12 M	≤1 Y	Idiopathic/S2–S3)	0.05%/15 s	NA	glucose and BSS
Schaal S [30], 2009	240(90∶66)	69 (55–86)/63 (54–78)	NA	20/100/20/100	≥1 Y	>1 Y	Idiopathic/(S2–S4)	0.5%/NA	270	sterile water
Nakamura Y [31], 2007	75(16∶38)	64.5±1.4/64.5±0.8	6∶10/12∶26	0.81±0.07/0.82±0.05	3 Y	2.6±0.35/2.8±0.39 M	Idiopathic/(S2–S4)	0.25%/IM	NA	NA
Thompson JT [32], 2007	123(62/32)	66.97/70.34	NA	20/160-2/20/125-2	≥1 Y	<2 Y	Idiopathic/(S2–S4)	0.05%/60 s	NA	NA
Nomoto H [33], 2008^c^	67(27∶40)	65.8±7.0/61.7±9.3	5∶22/14∶26	0.81±0.4/0.78±0.3	1 Y	NA	Mixed/(S2–S4)	0.25%/NA	NA	NA
Nagai N [34], 2007	53(35∶18)	65.3±6.6/64.3±5.5	8∶27/6∶12	0.83±0.27/0.89±0.23	>1 Y	3.9±3.8/4.7±5.5	Idiopathic/(S2–S4)	NA	NA	distilled water and BSS
Beutel J [35], 2007	40(19∶19)	67.2±4.7/69.3±5.9	7∶13/9∶11	20/40∶20∶50	6 M	NA	Idiopathic/(S2–S4)	0.005%/IM	NA	glucose
Ferencz M. [36], 2006	30(21/9)	65.7±5.8/70.0±4.9	7∶14/2∶7	0.89±0.23/0.80±0.21	20 M	6.4±4.5/7.2±4.3 M	NA/(S2–S4)	0.125%/IM	270	distilled water and BSS
Kumagai K [37], 2006	190(96/94)	65.3±7.3/65.3±6.7	33∶63/28∶66	0.70±0.34/0.78±0.33	26.2/30.7 M	2.9∶3.5 M	Idiopathic/(S2–S4)	0.1%/IM	NA	distilled water and BSS
Husson-Danan A [38], 2006	23/15	69.3±6.8/60.3±6.8	NA	NA	8.4±5.8/13.9±12.5 M	11.2±12.2/5.4±3.6 M	NA/(S2–S3)	0.05%/<30 s	NA	glucose
Lee KL [39], 2005^b^	37(19∶18)	70.7/68.6	NA	0.91/0.85	10.6/9.4 M	317.3/258.8 D	NA/(S2–S4)	0.05%–0.5%/IM	242/295	BSS
Karacorlu M [40], 2005^c^	30(15/15)	64.6/64.5	8∶7/9∶6	NA	7.2±1.5/6.4±2.7 M	<6 M	Idiopathic/(S3–S4)	0.05%/10 s	NA	NA
Slaughter K [41], 2004	68(34/34)	67.5/66.2	10∶24/9∶25	6∶26/6∶60	25/53 W	<1 Y	Mixed/NA	NA/30 s	NA	NA
Ando F [16], 2004	97(28∶21)	64.5/65.3	8∶20/7∶14	0.77±0.35/0.98±0.43	17.5/15.6 M	3.2/3.3 M	Idiopathic/(S2–S4)	0.5%/IM	NA	BSS
Lochhead J [15], 2004	68(34∶34)	69.9/67.5	10∶24/10∶24	1.00/0.99	7.7/6.3 M	<1 Y	NA/(S3–S4)	0.5%/10 s	NA	BSS
Sheidow TG [42], 2003	176(35∶44)	69.8/67.7	4∶31/15∶29	NA	14/9 M	3∶3.2 M	Idiopathic/(S2–S4)	0.5%/30 s	NA	BSS
Horio N [43], 2004	40(20∶20)	64.7±6.9/63.5±6.9	7∶12/5∶15	0.92±0.25/0.92±0.24	18.7±5.9/17.0±4.5 M	3.8±3.1/4.1±4.2 M	Idiopathic/(S2–S4)	0.125%/10–30 s	270	BSS
Eiko Tsuiki [25], 2007	140(96∶44)	65.9(18–97)	49∶91	NA	NA	NA	NA/NA	0.25%/IM	NA	NA
Kouki Fukuda [6], 2011^a^	53(22∶31)	68/67	12∶10/14∶17	0.59±0.27/0.61±0.29	>6 M	3∶3 M	Idiopathic/(S2–S4)	0.125%/IM	NA	BSS
Mavrofrides E [26], 2006	173(83∶90)	65.5±11.0/66.7±12.4	28∶49/29∶58	NA	9.0/9.5 M	5.0±6.2/5.2±6.1 M	Mixed/NA	0.25%/10 s	NA	BSS

— = no data provided; NA = not available ; RCT = randomized-controlled trials; LOE = level of evidence; BSS: balanced saline solution.

a The part included in the partly retrospective study is retrospective.

b The study quality is evaluated by Downs and Black score and Newcastle-Ottowa Scale (NOS). The Downs and Black score for both RCT and non-RCT while NOS for RCT only.

c The matching factors are: (1) age, (2) gender, (3) macular hole type, (4) symptom duration, (5) preoperative visual acuity, (6) one surgeon, (7), follow-up time.

**Table 3:** Description of the outcomes of interest in each trial.

	Anatomical Outcomes (Closure Rate)		Functional Outcomes (Visual Acuity)			Complications		
	Primary	Secondary	Gain≥20/40	Improved≥2 Lines	Increased LogMAR	RPE Changes	Retinal Detachment	Visual Field Defect
Brockmann T [27], 2011	12∶13/12∶12	NA	NA	NA	NA	NA	NA	NA
Shukla D [28], 2011	NA	13∶15/34∶35	1∶15/11∶35	NA	NA	1∶15/0∶35	No	NA
Christensen UC [29], 2009	32∶34/16∶18	33∶34/18∶18	24∶31/12∶17	NA	NA	14∶33/15∶17	1∶33/1∶17	1∶13/1∶17
Schaal S [30], 2009	NA	82∶90/50∶66	NA	69∶90/43∶66	NA	0∶90/0∶66	1∶90/1∶66	NO
Nakamura Y [31], 2007	16∶16/38∶38	NA	NA	NA	0.49±0.05/0.61±0.05	NA	NA	NA
Thompson JT [32], 2007	61∶62/31∶32	61∶62/32∶32	18∶62/11∶32	NA	NA	0∶62/0∶32	NO	No
Nomoto H [33], 2008	NA	27∶27/39∶40	16∶27/33∶40	22∶27/37∶40	0.47±0.35/0.58±0.30	NO	NO	NA
Nagai N [34], 2007	34∶35/17∶18	35∶35∶18∶18	19∶34/9∶16	NA	0.50±0.26/0.52±0.36	6∶35/2∶18	2∶35/0∶18	7∶35/0∶18
Beutel J [35], 2007	16∶19/16∶19	18∶19/19∶19	NA	11∶19/10∶19	NA	1∶19/0∶19	1∶19/1∶19	NA
Ferencz M [36], 2006	NA	NA	NA	NA	0.25±0.20/0.37±0.18	NA	NA	NA
Kumagai K [37], 2006	NA	95∶96/93∶94	83∶96/84∶94	78∶96/83∶94	NA	2∶96/1∶94	NA	NO
Husson-Danan A [38], 2006	NA	17∶23/15∶15	NA	NA	0.26±0.42/0.42±0.36	2∶20/3∶15	NA	1∶14/2∶18
Lee KL [39], 2005	NA	17∶19/17∶18	6∶14/11∶16	9∶14/13∶16	0.30±0.27/0.52±0.27	NA	3∶19/0∶18	NA
Karacorlu M [40], 2005	15∶15/15∶15	NA	NA	NA	0.11±0.06/0.12±0.05	NA	1∶15/0∶15	NA
Slaughter K [41], 2004	NA	31∶34/33∶34	7∶34/8∶34	NA	NA	No	0∶34/1∶34	NA
Ando F [16], 2004	NA	28∶28/18∶21	NA	NA	0.076±0.424/0.550±0.416	No	NA	8∶28/0∶21
Lochhead J [15], 2004	31∶34/25∶34	32∶34/31∶34	NA	13∶34/13∶34	NA	NA	NA	NA
Sheidow TG [42], 2003	34∶35/43∶44	NA	NA	25∶35/34∶44	NA	NA	NO	NA
Horio N [43], 2004	NA	20∶20/20∶20	NA	20∶20/20∶20	0.25±0.23/0.10±0.22	No	NA	NA
Eiko Tsuiki [6], 2007	NA	NA	NA	NA	NA	NA	NA	NA
Kouki Fukuda [6], 2011	NA	22∶22/31∶31	NA	NA	0.45±0.24/0.51±0.26	NA	NO	NA
Mavrofrides E [26], 2006	NA	67∶77/72∶87	NA	NA	NA	NA	NA	NA

NA = not available; No = no related complication in both case group and control group; RPE = retinal pigment epithelium.

### 4. Efficacy analysis

#### 4.1 Main results

The main results of the meta-analysis are shown in Table 4. Among all of the included outcomes, the rate of VA gain ≥20/40 (OR, 0.65; 95% CI, 0.43 to 0.97; *P* = 0.033), increased LogMAR (WMD, −0.09; 95% CI, −0.16 to −0.02; *P* = 0.011) and visual field defects (OR, 4.27; 95% CI, 1.34 to 13.63; *P* = 0.014) significantly differed between the ICG group and the non-ICG group. No significant differences were detected between the two groups for the anatomical outcomes, the remaining functional outcomes or most of the postoperative complications. Significant interstudy heterogeneity was observed in VA improvement (I^2^, 81.3; *P*<0.001), increased LogMAR values (I^2^, 80.8; *P*<0.001), RPE changes (I^2^, 51.6; *P* = 0.006) and optic nerve fiber layer changes (I^2^, 81.7; *P* = 0.004).

**Table 4:** Description of the main results of meta-analysis.

Outcome of Interest	No. of Studies	No. of Eyes		Overall Effect		Study Heterogeneity	*p* Value
		ICG Group	non-ICG Group	WMD/OR (95% CI)	*p* Value	I^2^, %	
Anatomical outcome							
Rate of primary closure	7	232	177	1.76 (0.81 to 3.80)	0.153	0.0	0.833
Rate of secondary closure	13	557	513	1.30 (0.83 to 2.05)	0.258	20.6	0.235
Rate of final closure	15	714	691	1.21 (0.78 to 1.88)	0.388	9.4	0.348
Functional Outcome							
Rate of VA≥20/40	8	313	284	0.65 (0.43 to 0.97)	0.033	0.0	0.496
Rate of VA improved≥2 Lines	6	296	294	0.88 (0.60 to 1.31)	0.532	29.5	0.214
Rate of VA improved	5	168	166	1.09 (−0.69 to 1.74)	0.704	81.3	<0.001
Increased LogMAR	10	225	223	−0.09 (−0.16 to −0.02)	0.011	44.0	<0.001
Complications							
Risk of RPE change	6	177	218	0.63 (0.31 to 1.30)	0.209	51.6	0.066
Risk of retinal detachment	7	245	187	1.48 (0.54 to 4.08)	0.451	0.0	0.776
Risk of retinal tears	3	138	127	1.12 (0.49 to 2.54)	0.791	34.9	0.215
Risk of visual field defect	4	90	74	4.27 (1.34 to 13.63)	0.014	27.7	0.246
Risk of macular edema	2	52	50	1.10 (0.12 to 9.90)	0.933	0.0	0.742
Risk of ONFL change	3	80	53	1.21 (0.10 to 14.73)	0.880	81.7	0.004

VA: visual acuity; OR: odds ratios; WMD: weighted mean difference; ICG: indocyanine green; RPE: retinal pigment epithelium; ONFL: optic nerve fibres layer.

**Figure 2:**
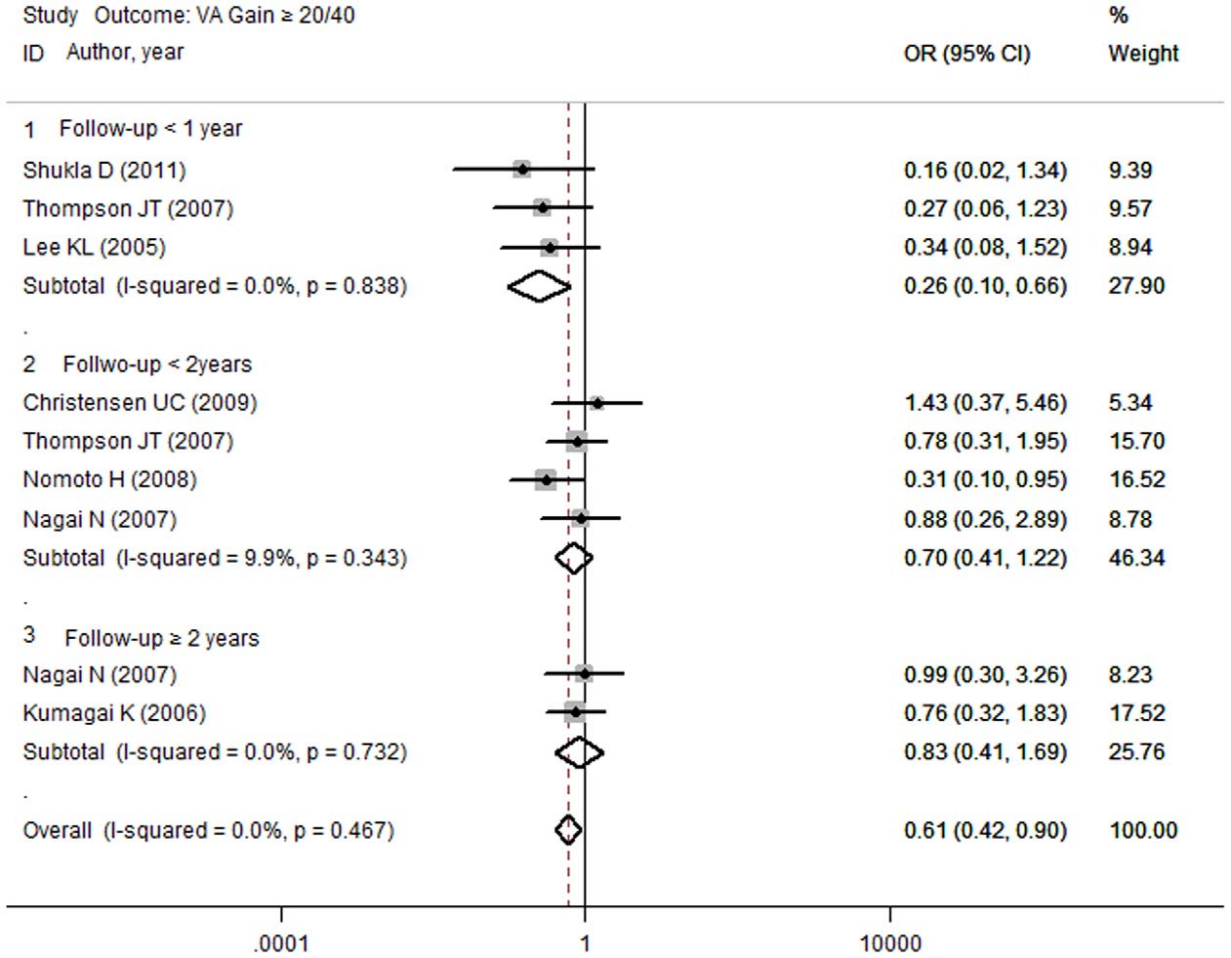
Forest plot for VA gain ≥20/40 in different follow up duration. The rate of VA gain ≥20/40 was lower in ICG group compared with the non-ICG group, while no difference was observed in longer follow-up duration.

Because the results of the meta-analysis showed relatively less VA improvement in the ICG group, VA improvement was analyzed at 1 year, 2 years and 3 years of follow up. Figure 2 shows that, within 1 year, the incidence of VA gain over 20/40 was lower in the ICG group than in the non-ICG group (OR, 0.26; 95% CI, 0.10 to 0.66; *P* = 0.005). However, after 2 years (OR, 0.70; 95% CI, 0.41 to 1.22; *P* = 0.211) and 3 years (OR, 0.83; 95% CI, 0.41 to 1.69; *P* = 0.610), no difference was observed in the incidence of VA gain. No difference was observed in the rate of VA improvement ≥2 lines and increased LogMAR at the different follow-up durations.

#### 4.2 Subgroup analysis

**Figure 3:**
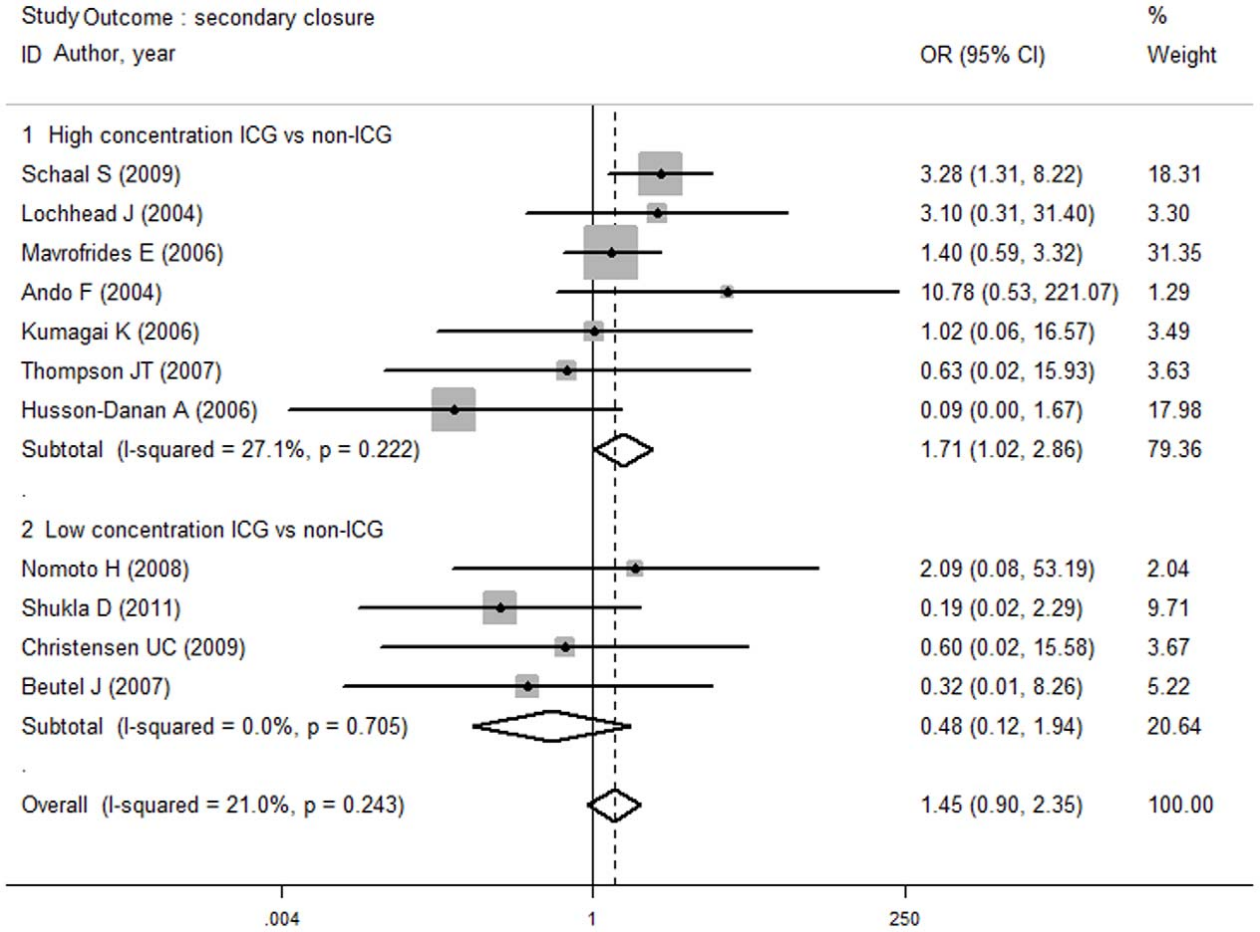
Subgroup analysis rate of secondary closure high and low concentration of ICG versus non-ICG group. Better secondary closure rate was detected in high concentration ICG compared with non-ICG group, while no difference was observed in low concentration ICG compared with non-ICG group.

A subgroup analysis of the anatomical outcomes was conducted using different control groups and different ICG concentration groups. The primary closure rate did not differ between the ICG group and the unstained group (OR, 2.53; 95% CI, 0.90 to 7.17; *P* = 0.080) or the other stains group (OR, 1.06; 95% CI, 0.32 to 3.53, *P* = 0.925). There was no difference in the rate of primary closure between the high-concentration ICG (OR, 2.75, 95% CI 0.81 to 3.51; *P* = 0.106) or low-concentration ICG (OR, 1.16; 95% CI, 0.39 to 3.51; *P* = 0.789) groups and the non-ICG group. No difference in the secondary closure rate was shown between the ICG group and the unstained group (OR, 1.55; 95% CI, 0.94 to 2.54; *P* = 0.247) or between the ICG group and the other stains group (OR, 0.49; 95% CI, 0.14 to 1.64; *P* = 0.086). A high concentration of ICG resulted in higher secondary closure rates (OR, 1.71; 95% CI, 1.02 to 2.86; *P* = 0.012), but no difference was observed between the low ICG concentration group and the non-ICG group (OR, 1.45; 95% CI, 0.12 to 1.94; *P* = 0.093) (Figure 3).

**Figure 4:**
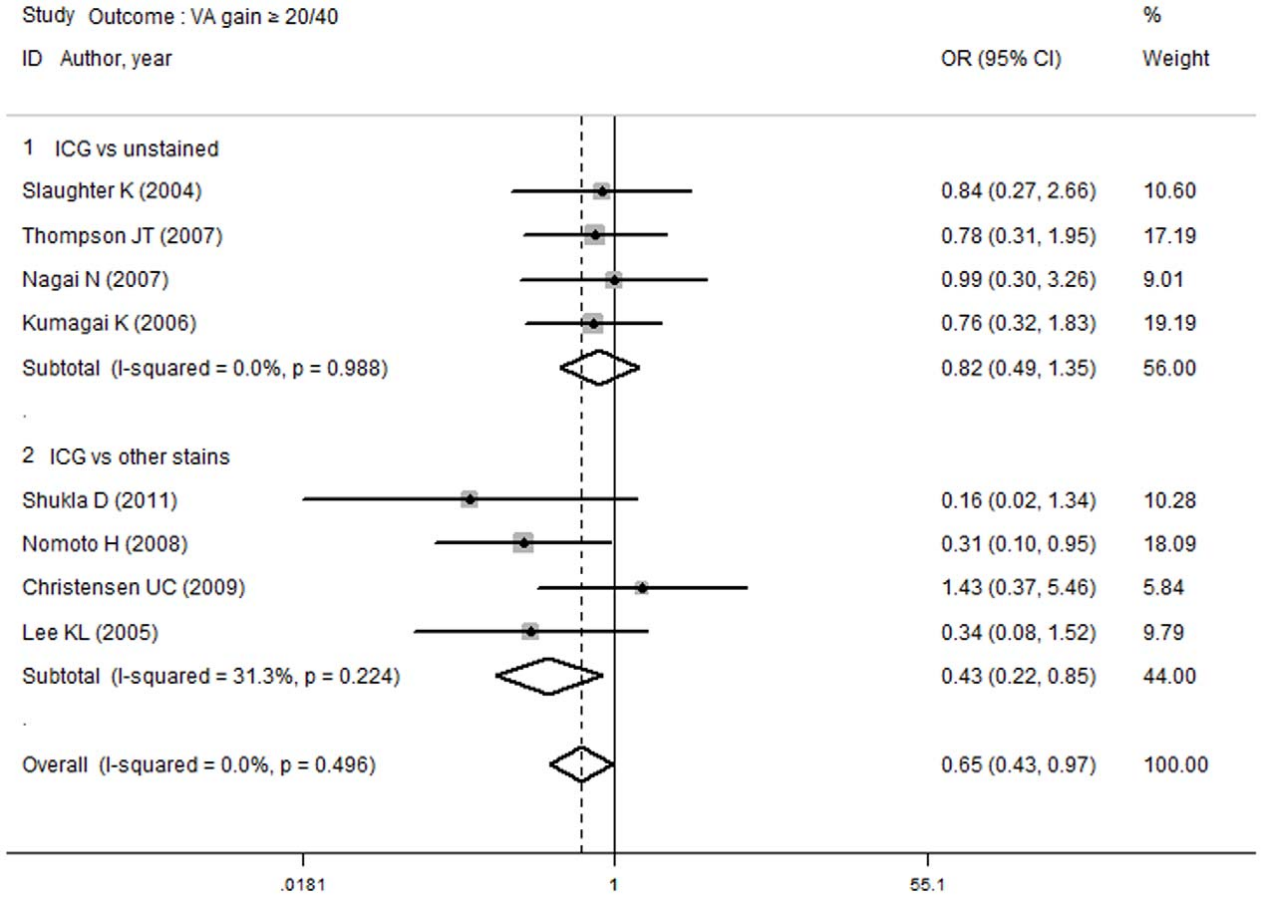
Subgroup analyses of outcome VA gain 20/40 in ICG group versus unstained and other stains groups. The lower rate of VA gain ≥20/40 was observed in ICG group compared with other stains group while no difference was observed in ICG group compared with unstained group.

**Figure 5:**
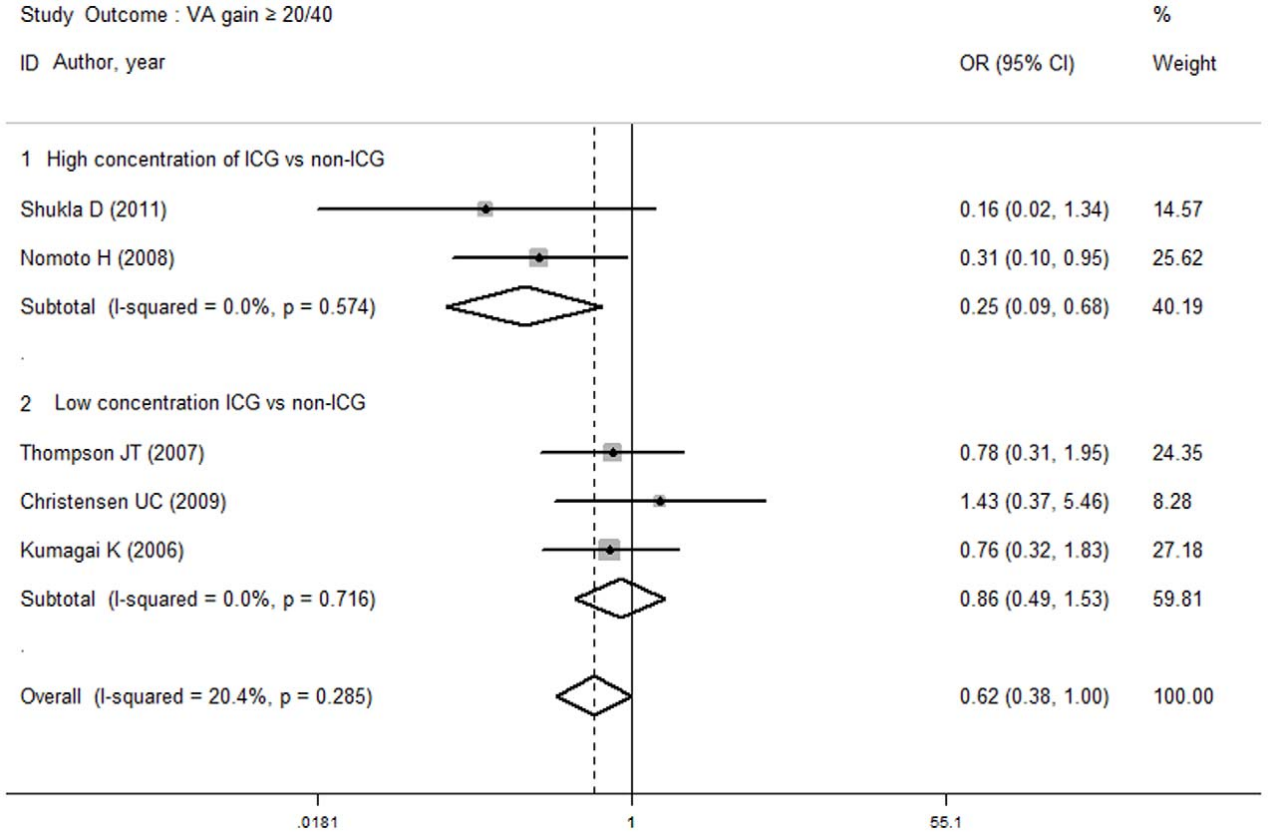
Subgroup analyses of outcome VA gain 20/40 in high and low concentration of ICG versus non-ICG group. The rate of VA gain ≥20/40 was lower in high concentration ICG group compared with non-ICG group, while no difference was observed between low concentration ICG and non-ICG group.

The functional outcomes, including the rates of VA gain ≥20/40, VA improvement ≥2 lines and increased LogMAR, were included in an additional subgroup analysis. The rate of VA improvement ≥2 lines did not differ in the subgroup analysis between the ICG group and the unstained or other stains groups. Meanwhile, no difference was observed between the high or low ICG concentrations and the non-ICG group. Figure 4 demonstrates that the incidence of VA gain ≥20/40 in the ICG group was less than in the other stains group (OR, 0.43; 95% CI, 0.22 to 0.85; *P* = 0.015), but no difference was observed between the ICG group and the unstained group (OR, 0.82; 95% CI, 0.49 to 1.35; *P* = 0.435). As shown in Figure 5, the rate of VA gain ≥20/40 in the high ICG concentration group was lower than in the non-ICG group (OR, 0.25; 95% CI, 0.09 to 0.68; *P* = 0.006). However, no difference was found between the low ICG concentration group and the non-ICG group (OR, 0.86; 95% CI, 0.49 to 1.53; *P* = 0.608). In the increased LogMAR outcome subgroup analysis, no difference was observed between the ICG and unstained groups (WMD, −0.11; 95% CI, −0.23 to 0.02; *P* = 0.096) or between the ICG and other stains groups (WMD, −0.07, 95% CI, −0.16 to 0.01; P = 0.099). Additionally, no difference was found between the high-concentration (WMD, −0.10; 95% CI, −0.21 to 0.01; *P* = 0.063) or low-concentration (WMD, −0.03; 95% CI, −0.14 to 0.07; *P* = 0.547) ICG group and the non-ICG group.

**Figure 6:**
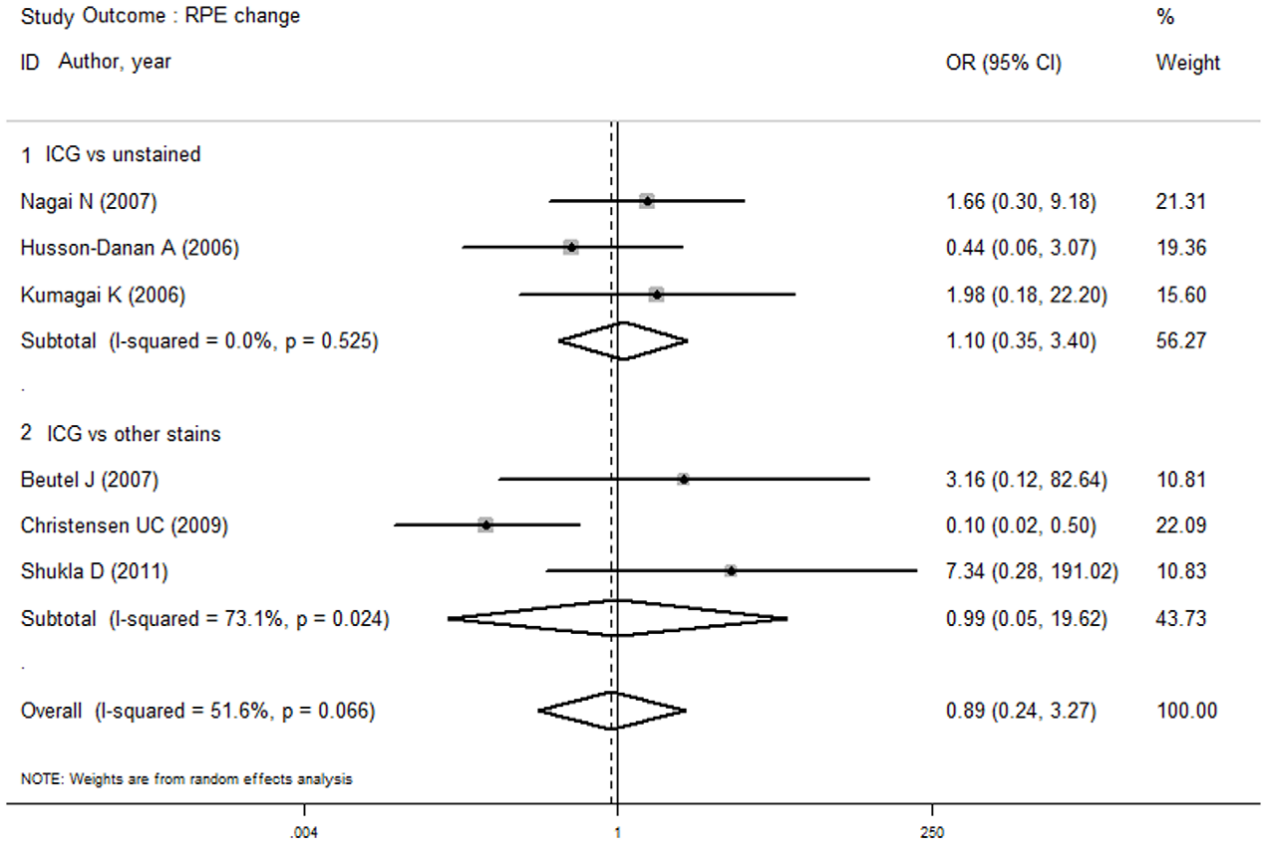
Subgroup analyses of outcome RPE change in ICG group versus unstained and other stains groups. There were no differences in the risk of RPE changes between the ICG group and the unstained or other stains groups.

**Figure 7:**
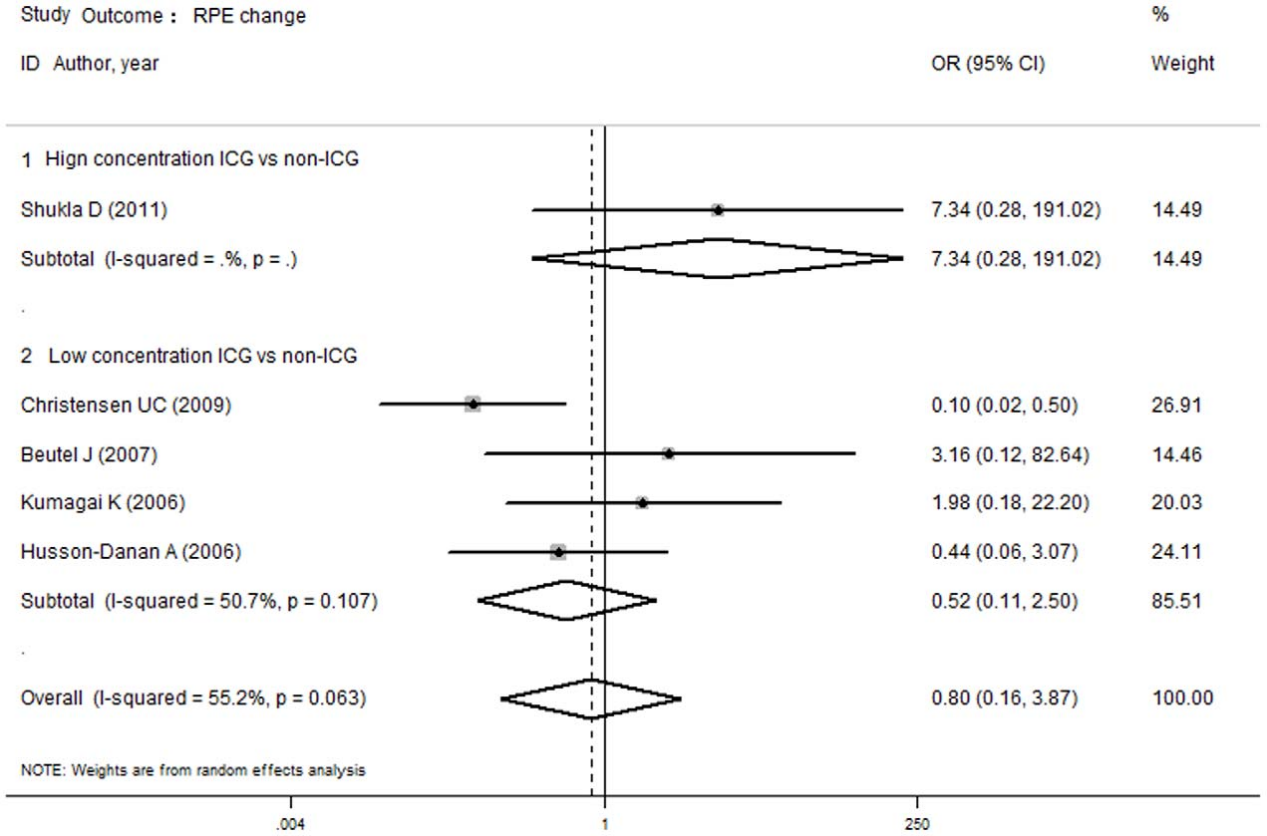
Subgroup analyses of outcome VA gain 20/40 in high and low concentration of ICG versus non-ICG group. There were no differences between the high or low concentration of ICG group and the non-ICG group.

According to the above results, no difference was established in the risk of RPE changes between the ICG and non-ICG groups. To evaluate the influence of different control group conditions (unstained and other stains) and different ICG concentrations (high and low concentrations), a subgroup analysis was conducted. The analysis found no difference in the risk of RPE changes between the ICG group and the unstained or other stains groups, and there was no difference between the high or low concentration of ICG group and the non-ICG group. Figures 6 and 7 depict the forest plot of the subgroup analysis for secondary closure.

### 5. Heterogeneity, sensitivity analysis and publish bias

Some of the outcomes of interest displayed great heterogeneity in the meta-analysis results (Table 4). The heterogeneity of VA increased LogMAR unit was significant and dropping included study by hand, subgroup analysis and meta regression didn't provide good results. However, the studies included in the analysis of RPE changes were also heterogeneous, and the interstudy heterogeneity was not significant after dropping the most weighted study [29] (I^2^ = 0.0%, *P* = 0.610).

A sensitivity analysis was conducted after 3 RCTs [29], [35], [43] were excluded, and the results are presented in Table 5. No contradictory significant differences were observed in the results of the sensitivity analysis compared to the previous analysis. We therefore performed a heterogeneity interstudy, and the result indicated confidence in the conclusions of this study. An assessment using the Begg rank correction and the Egger liner regression test demonstrated no evidence of publication bias.

**Table 5:** Result of sensitivity analysis about trial types for outcome of interest.

Outcome of Interest	No. of Studies	No. of Eyes		Overall Effect		Study Heterogeneity	*p* Value
		ICG Group	non-ICG Group	WMD/OR (95% CI)	*p* Value	*I^2^*, %	
Anatomical outcome							
Rate of primary closure	5	179	140	2.02 (0.78 to 5,26)	0.148	0.0	0.681
Rate of secondary closure	11	504	476	1.37 (0.86 to 2.19)	0.186	28.7	0.172
Rate of final closure	13	553	532	1.27 (0.81 to 1.99)	0.297	17.2	0.271
Functional Outcome							
Rate of VA≥20/40	7	282	267	0.60 (0.39 to 0.91)	0.017	0.0	0.543
Rate of VA improved≥2 Lines	6	296	294	0.88 (0.60 to 1.31)	0.532	29.5	0.214
Rate of VA improved	5	168	166	1.09 (−0.69 to 1.74)	0.704	81.3	<0.001
Increased LogMAR	9	205	203	−0.12 (−0.18 to 0.18)	0.001	75.9	<0.001
Complications							
Risk of RPE change	4	166	162	o.90 (0.24 to 3.27)	0.862	51.6	0.066
Risk of retinal detachment	5	193	151	1.89 (0.57 to 6.30)	0.299	0.0	0.628
Risk of retinal tears	2	125	11 o	0.76 (0.30 to 1.94)	0.571	0.0	0.891
Risk of visual field defect	3	77	57	5.19 (1.40 to 19.25)	0.014	45.1	0.162
Risk of macular edema	2	52	50	1.10 (0.12 to 9.90)	0.933	0.0	0.742
Risk of ONFL change	2	47	36	0.95 (0.36 to 2,50)	0.919	89.9	0.002

VA: visual acuity; OR: odds ratios; WMD: weighted mean difference; ICG: indocyanine green; RPE: retinal pigment epithelium; ONFL: optic nerve fibres layer.

## Discussion

The current meta-analysis summarized the anatomical and functional outcomes and the postoperative complications of ICG-stained ILM peeling with a total of 858 case eyes and 727 control eyes. The results indicated that the rate of VA and the risk of postoperative visual field defects were worse in the ICG group than in the non-ICG group. However, the anatomical outcomes and other postoperative complications were similar (*P*>0.05). The additional analyses showed that VA improvement over varied follow-up durations and that the VA of the ICG group was worse only within the first postoperative year; no difference existed at later follow-up dates. No differences were observed between the ICG group and the unstained group. However, the postoperative VA in the ICG group was worse compared with that in the other stains group. Although the anatomical and functional outcomes were not significantly different between the low ICG concentration group and the non-ICG group, the high ICG concentration group showed a better secondary closure rate but a worse VA outcome.

Since Kim first proposed the use of ICG to stain the ILM for better visualization and to assistant with ILM removal in 1999 [1], ILM peeling and ICG staining have been regarded as potential methods for improving anatomical and functional outcomes [44]. However, the use of ICG in MH surgery was followed by reports of postoperative adverse effects [45], and no concordant conclusion about the value of ICG was achieved. In 2008, Rodrigues and collaborators [46] conducted a meta-analysis to evaluate ICG in the management of MHs and reached the conclusion that ICG-assisted ILM peeling was related to no differences in the closure rate, a worse VA and an increased risk of RPE changes. However, because the included studies had a cut-off date of June 2004 and the studies included were not directly comparative studies, our updated meta-analysis was of certain importance.

The anatomical outcomes of the ICG group and non-ICG group were not significantly different, regardless of the use of other stains in the control group. In a retrospective multicenter study including 1627 eyes, patients in whom ICG was used to stain the ILM had a lower percentage of anatomical success [1]. However, the study was limited because its aim was to compare MH surgery with and without ILM peeling and the use of ICG was not standardized. Meanwhile, there were also reports indicating no innocuous or helpful effects of ICG use for MH surgery [6], [15], [40]. In this meta-analysis, no differences were identified following the use of ICG in MH surgery. However, relatively high concentrations (over 0.1%) were found to correlate with a better secondary closure rate. Kwok and colleagues [47] reported that 0.125% ICG resulted in significantly better ILM staining than concentrations of 0.025% to 0.05% ICG, but no difference in peeling time was observed. It is possible that a higher concentration of ICG increased the visibility of the ILM and improved the rate of complete ILM peeling, avoiding secondary adjunct use of ICG.

Similar to the previous meta-analysis [48], worse functional outcomes were also detected in this study. Since ICG was first used in MH surgery, significantly worse visual outcomes have been reported by several case series and clinical trials [1], [16], [34], [42]. The increased postoperative complication rate may have resulted in a lower VA improvement [45]. Another hypothesis was that a deeper cleavage plane moved to the innermost layers after the application of ICG [49]. In additional analyses, a significant difference in VA improvement was found between ICG use and the use of other stains, while in our study, no significant differences were found between ICG use and no ICG. The use of ICG did not decrease VA improvement, perhaps because of the advanced technique of the surgeons and increased experience with the application of ICG [50]. Compared with the other stains group, worse functional outcomes were found in the ICG group. Meanwhile, ICG use did not result in better anatomical outcomes when compared to the use of other stains, and it was a great challenge to the importance of ICG in ILM peeling. In the present study, a higher ICG concentration was correlated with less VA improvement, and this was in agreement with reports demonstrating that a lower ICG concentration provided a better VA outcome [51], [52]. However, even though worse functional outcomes were observed over a short-term follow up in the ICG group, the long-term follow up demonstrated no difference between the ICG group and the non-ICG group. Similar long-term VA outcome following ILM peeling with and without ICG were previously reported by several studies [34], [37].

The results failed to demonstrate a relationship between ICG application and RPE changes. The causes of RPE changes were described as follows: 1) ICG was the result of direct toxicity to the RPE; 2) ICG enhanced phototoxicity to the RPE [11]. Burk and associates [53] applied 0.5% ICG during autopsies and observed no effects on the RPE. RPE changes were observed after a 3-day exposure period in a rabbit model [54]. The application of ICG in cultured human RPE cells resulted in decreased mitochondrial enzyme activity, while no cellular morphological or ultrastructural changes were observed [13]. Another in vitro experiment showed that ICG was toxic to cultured RPE cells following exposure to concentrations between 0.5% and 0.05% for 3 minutes; no toxicity was observed with trypan blue. [55]. Several reports have demonstrated that lower ICG concentrations, shorter exposure times and appropriate light use were possible methods of decreasing the toxicity of ICG [50], [55], [56]. In this meta-analysis, after excluding the studies demonstrating no RPE changes in both the ICG and non-ICG groups, the earliest included studies were published in 2006 [37], [38]. The resulting increased experience with ICG application is a possible explanation for the different results obtained in the present study when compared with a previous meta-analysis [48].

With the exception of RPE changes, several postoperative complications are thought to be associated with ICG use, including visual field defects, a reduced rate of MH closure, ICG persistence in the retina and optic nerve, optic atrophy, macular edema and retinal tears [50]. In this meta-analysis, only visual defects were observed to be significantly different. Although visual field defects have been considered common in MH surgery with and without the use of ICG [57], several authors have reported an increased rate of visual field defects in ICG-stained eyes [25], [58]. The application of fluid-air [59] was regarded as a common explanation for the visual field defects, and potential toxicity of ICG to the optic nerve was also reported [60]. However, as the techniques of the surgeons and their experience with ICG application increased, this adverse effect was possible to avoid.

In the included studies, 4 studies [30], [36], [39], [43] reported the osmolarity values (242 to 295 mOsm), while the most common osmolarity was 270 mOsm. A total of 14 studies reported the solvent used. Stalmans et al [61] reported that hypo-osmolar solutions resulted in higher cell death, while the iso-osmolar solutions was not related with RPE cell survival and it was the hypo-osmolarity rather than ICG itself related to the toxicity. However, another study [62] reported that hypo-osmolarity alone didn't produce toxicity, while it produced cell damage when low osmolarity was combined with ICG. There was no accordant opinion in the relationship between osmolarity and ICG toxicity and more studies are required. Glucose 5% and BSS are solvent usually used and both of them might result in damaging effect [63], [64]. Compared with ICG diluted with BSS, ICG diluted with glucose 5% produced a shift of the absorption band toward longer wavelengths [65] and there was a hypothesis that the light-absorbing proprieties of dye altered by glucose 5% would reduce the photosensitivity; however, no definitive evidences to prove this existed by now.

The strengths of current meta-analysis were as follows. First, the relatively high number of the included studies and cases provided a better power for the analysis. Second, the consonance of the previous results and the sensitivity analysis demonstrate that the conclusions from this analysis were robust. Despite these advantages, some limitations of the current study should not be ignored. First, this study was limited by the low quality of the retrospective studies included and the lack of RCT-based evidence. It's hard to conduct a meta-analysis in surgical practice and the main challenges of observational studies included selection bias, confounding bias known or unknown, and reporting bias. Second, the symptom duration and MH stage in each trial were not perfectly matched, which may also influence the outcomes of interest. Third, some parameters of interest demonstrated a large degree of heterogeneity. Some were explained, but the heterogeneity of the increased LogMAR was not explained. This may be the result of different surgical techniques or different methods of measuring the LogMAR VA in different trials.

Leaving the limitations aside, we believe that the results of the current meta-analysis are credible. Because the anatomical and functional outcomes of the ICG-stained group were not better, there is no evidence of clinical superiority of ICG use in MH surgery. Because ICG resulted in less VA improvement than other stains group, such as trypan blue, the toxicity of ICG should be considered when choosing the various staining methods.
